# Amelioration on oxidative stress, testosterone, and cortisol levels after administration of Vitamins C and E in albino rats with chronic variable stress

**DOI:** 10.14202/vetworld.2021.137-143

**Published:** 2021-01-18

**Authors:** Nanik Hidayatik, Agus Purnomo, Faisal Fikri, Muhammad Thohawi Elziyad Purnama

**Affiliations:** 1 Department of Veterinary Clinical Pathology and Physiology, Faculty of Veterinary Medicine, Universitas Airlangga, Surabaya, Indonesia; 2 Department of Veterinary Surgery and Radiology, Faculty of Veterinary Medicine, Universitas Gadjah Mada, Yogyakarta, Indonesia; 3 Department of Veterinary Anatomy, Faculty of Veterinary Medicine, Universitas Airlangga, Surabaya, Indonesia

**Keywords:** chronic variable stress, oxidative stress parameters, Vitamin C, Vitamin E

## Abstract

**Background and Aim:**

Stress can cause physiological and biological disorders in the body. On the other hand, antioxidants from vitamins and minerals are effective for stress treatment. Therefore, this study aimed to evaluate the effect of the administration of Vitamins C and E on serum superoxide dismutase (SOD), malondialdehyde (MDA), catalase (CAT), glutathione peroxidase (GPx), testosterone, and cortisol activity in albino rats with chronic variable stress (CVS).

**Materials and Methods:**

Twenty albino rats were randomly assigned into four treatment groups: C was administered normal saline; T1 was administered Vitamins C and E; T2 was only induced CVS; and T3 was induced CVS followed by Vitamins C and E administration. All treatments were applied for 4 weeks, respectively. Furthermore, 5 mL of blood samples were collected intracardially. Body weight data were collected for the initial and final weights. From serum samples, SOD, GPx, and CAT were measured using the enzymol method; MDA was measured using the high-performance liquid chromatography method; and testosterone and cortisol were measured using the enzyme-linked immunosorbent assay method. All variables were analyzed statistically using analysis of variance followed by the Duncan test (p<0.05).

**Results:**

Our findings showed that the T1 and T3 groups significantly decreased (p<0.001) compared to T2 in the following parameters: SOD, MDA, GPx, and cortisol. Meanwhile, CAT and testosterone levels in the T1 and T3 groups were significantly increased (p<0.001) compared to the T2 group. In addition, the weight gain in T1 and T3 groups was significantly increased (p<0.001) compared to T2 group.

**Conclusion:**

It can be concluded that the administration of Vitamins C and E had a significant effect to alleviate SOD, MDA, GPx, and cortisol and to improve the testosterone level in albino rats with CVS.

## Introduction

Stress can be categorized as acute or chronic, depending on the duration of the exposure. Both acute and chronic stresses are known as a major cause of physiological and biological disorders, especially due to their significant impact on the production of free radical or reactive oxygen species (ROS) [1-3]. The production of free radicals is influenced by external factors such as heat, trauma, noise, infection, radiation, hyperoxia, toxins, and physical exercise [4]. It is well known that normal cells produce small amounts of ROS as essential substances in the body, but their accumulation can damage macromolecules such as fats, proteins, carbohydrates, and DNA structures [4-7]. In conditions of stress, it has been shown that there are increased antioxidant enzymes activities, including superoxide dismutase (SOD), catalase (CAT), and glutathione peroxidase (GPx) [4,8]. In addition, cortisol is known as one of the primary hormones that define stress conditions, as it has been reported that it increases during stress conditions [9-11] and will be affected by the decreased testosterone level. Testosterone is an active hormone that regulates libido and supports spermatid elongation during spermiogenesis [12,13]. As a result, stress has a negative impact on growth rate, feed consumption, body weight, libido, and animal production [14].

In the past decade, many studies were conducted using antioxidants from vitamins and minerals for stress treatment [5,15]. Vitamins C and E are known as non-enzymatic antioxidants that are effective for blocking the negative effects of oxidative stress [5]. Vitamin C can protect proteins, lipids, carbohydrates, and nucleic acids from damage by pro-oxidants generated during normal metabolism [16]. Vitamin E is the principal defense molecule against oxidant-induced membrane injury [5]. A combination of Vitamins E and C was reported to have decreased the effects of oxidative stress better than those of vitamins alone [17,18].

The aim of this present study that utilized a modified chronic variable stress (CVS) from Mueller and Bale [19] was to evaluate the SOD, malondialdehyde (MDA), CAT, GPx, testosterone, and cortisol activities after the administration of Vitamins C and E in albino rats.

## Materials and Methods

### Ethical approval

This study was approved by Animal Ethics No. 443/HRECC.FODM/VII/2019 of Universitas Airlangga.

### Study period and location

This study was conducted for 2 months (July and August 2019). Albino rats were reared in the Laboratory Animal Facility, Faculty of Veterinary Medicine, Universitas Airlangga. Laboratory examinations were performed at the Department of Veterinary Clinical Pathology and Physiology, Faculty of Veterinary Medicine, Universitas Airlangga and Gamma Scientific Biolab, Malang, East Java.

### Experimental design

To assess the impact of CVS in this study, the body weight of the albino rats was monitored before and after the study. A total of 20 male albino rats were randomly divided into four treatment groups with five replications, respectively: C was administered normal saline; T1 was administered Vitamins C and E; T2 was induced CVS; and T3 was induced CVS followed by Vitamins C and E administration.

The CVS was induced according to the Mueller and Bale method [19], but with a slight modification in terms of restraint, multiple cage, sleep deprivation, predator exposure, temperature stress, and noise (Table-1). Six painless, non-habituating stress models were performed, with the details as follows: Rats were restrained in a 900 cm^2^ enclosure area during the light cycle, multiple cage changes done during the light/dark cycle, and saturated beddings with water were placed overnight to cause sleep deprivation. Predator exposure was done by placing a cat in front of the enclosed cage with a wire mesh separation. Temperature stress was attained by setting it to 37°C. Finally, crowded exposure was conducted using 100 decibels of sound during the light cycle.

**Table-1:** The chronic variable stress modification procedure.

Stress type and schedule	Total hours in 4 weeks
Restraint, 20 min	9 h
Multiple cage, 20 min for 2 h	56 h
Sleep deprivation, 12 h	336 h
Predator exposure, 24 h	672 h
Temperature stress, 12 h	336 h
Noise, 4 h	112 h

After the respective stressor, rats were returned to their respective cages. Cages were cleaned and the litter was replaced before the rats were returned. The T2 and T3 groups were induced by CVS according to the aforementioned method. Furthermore, T1 and T3 groups were administered Vitamins C and E. A combination dose of Vitamin C (Bio C, Indonesia) 7 mg/kg and Vitamin E (Nutrilite™, Indonesia) 5 mg/kg was used. Vitamins C and E were dissolved in drinking water (0.1 mL/rats) and then administered orally after CVS induction. All treatments were applied for 4 weeks, respectively. After 4 weeks of treatment, all rats were sacrificed by cervical decapitation. Blood samples were collected intracardially for biochemical analysis.

### Serum evaluation

A total of 5 ml of the blood samples were collected intracardially. The blood sample was allowed to stand at room temperature for 15-30 min. Thereafter, it was centrifuged using centrifuge Hettich EBA 200^®^ (Hettich, Germany) at 4000 rpm for 15 min. The resulting supernatant designated serum was carefully aspirated using a Pasteur pipette into a microtube and stored at −4°C. Serum testosterone and cortisol were estimated using the enzyme-linked immunosorbent assay method (My-Bio-Source^®^, San Diego, CA, USA) [20]. The remaining serum was used to measure serum SOD, MDA, CAT, and GPx levels [21,22].

### Statistical analysis

Data were expressed as mean±standard deviation and analyzed statistically using a one-way analysis of variance followed by the Duncan test for comparisons between the groups. Differences were considered significant at p<0.05. The analysis was performed using SPSS v25 (IBM, USA).

## Results

### Body weight

The increase in body weight of the albino rats during the study was significantly different between the groups. The highest increase in body weight was observed in the T1 group, while the lowest final body weight was recorded in the T2 group, being approximately 59% and 5%, respectively. There was a significant increase in body weight in the T3 group (p<0.001) compared to the T2 group. On the other hand, there was a more significant increase in the T1 group (p<0.05) compared to the T3 group (Table-2). This study demonstrated an increase in the percentage of body weight increase in the Vitamins C and E groups compared to the group exposed to CVS.

**Table-2:** Body weight of the albino rats at the end of treatment.

Groups	Initial weight (g)	Final weight (g)	% increase in body weight
C (control)	153.0±1.58^a^	222.4±5.03^b,***^	45.4±4.28^b,***^
T1 (CE)	151.6±1.34^a^	240.2±7.69^a,***^	58.5±5.53^a,***^
T2 (CVS)	151.2±0.84^a^	158.4±6.35^c^	4.8±3.77^c^
T3 (CE+CVS)	151.0±1.00^a^	225.6±11.72^b,***^	49.4±7.34^b,***^

Values are expressed in mean±SD (n=5 animals for each four groups). Values are represented statistically ^a,b,c^when compared with C group value; *p<0.05, **p<0.01,

*** p<0.001, when compared with T2 group value. CVS=Chronic variable stress, SD=Standard deviation

### Antioxidant enzyme levels

**Figure-1:**
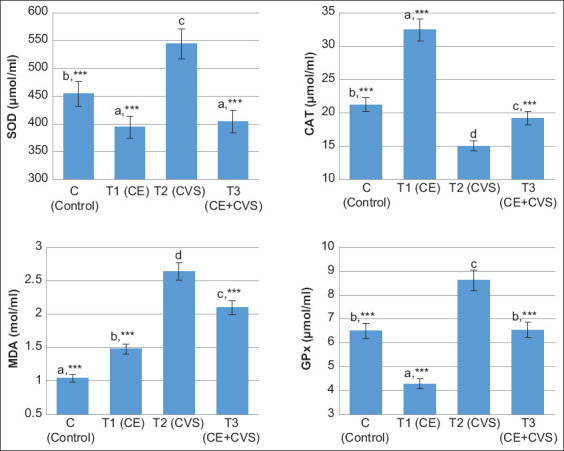
The activities of SOD, CAT, MDA, and GPx in serum after administration of Vitamins C and E in albino rats with chronic variable stress. Values are expressed in mean±standard deviation (n=5 animals for each four groups). Values are represented statistically ^a,b,c,d^when compared with C group value; *p<0.05, **p<0.01, ***p<0.001, when compared with T2 group value. SOD=Superoxide dismutase, CAT=Catalase, MDA=Malondialdehyde, GPx=Glutathione peroxidase.

The T1 and T3 groups showed significantly decreased (p<0.001) levels of SOD, MDA, and GPx compared to the T2 group. Furthermore, the groups administered with Vitamins C and E (T1 and T3) had lower SOD levels, but the levels were not significantly different between these groups (p>0.05). Regarding MDA and GPx, their levels were significantly lower in the T1 and T3 groups (p<0.001) compared to the T2 group. However, the T1 group showed the lowest levels compared to the T3 group. On the other hand, the levels of CAT in the T1 and T3 groups were significantly higher (p<0.001) compared to the T2 group (Figure-1). The current study revealed the role of Vitamins C and E in reducing the levels of SOD, MDA, and GPx, which are parameters of oxidative stress.

**Figure-2:**
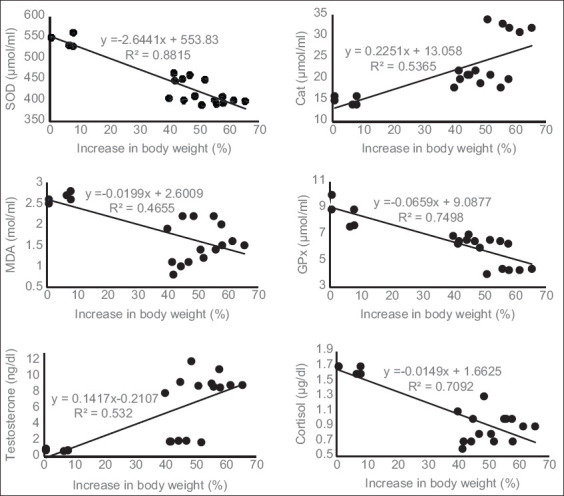
Regression plot for each parameter at increasing body weight.

Based on the regression analysis, the levels of SOD, MDA, and GPx had a negative correlation with increasing rat body weight (Figure-2). An increase in body weight was followed by a decrease in SOD, MDA, and GPx levels, with formulation; y=−2.6441x+553.83; y=−0.0199x+2.6009; and y=−0.0659x+9.0877, respectively. In contrast, CAT level had a positive correlation with increasing rat body weight (Figure-2). An increase in rat body weight was followed by an increase in CAT levels (y=0.2251x+13.058).

### Hormone levels

**Figure-3:**
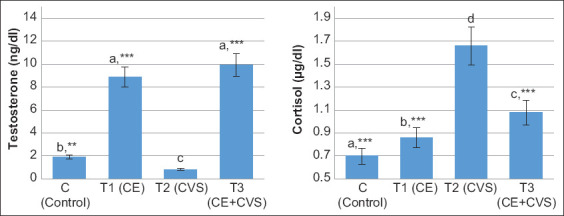
The level of testosterone and cortisol in serum after administration of Vitamins C and E in albino rats with chronic variable stress. Values are expressed in mean±standard deviation (n=5 animals for each four groups). Values are represented statistically ^a,b,c,d^when compared with C group value; *p<0.05, **p<0.01, ***p<0.001, when compared with T2 group value.

Vitamins C and E administration also led to an increase in testosterone levels and a decrease in cortisol levels. When compared to T2, the testosterone levels in the T1 and T3 groups were shown to be significantly increased (p<0.001). However, T1 and T3 showed no significant difference (p>0.05) (Figure-3). In the cortisol determination, all groups showed a significant decrease in cortisol levels (p<0.001) compared to the T2 group (Figure-3), thus proving that Vitamins C and E modulate stress reduction due to CVS.

The correlations of increased body weight with testosterone and cortisol levels were indicated to be positive and negative, respectively (Figure-2). Increased rat body weight was followed by an increase in testosterone levels (y=0.1417x−0.2107) and vice versa for cortisol levels (y=−0.0149x+1.6625).

## Discussion

In this study, we investigated the effect of combined treatment with Vitamins C and E on antioxidant enzymes activities and the levels of affected hormones in albino rats with CVS. Using these data, we obtained comparative information to elucidate the antioxidant effect of Vitamins C and E in CVS.

Vitamin C or E treatment alone was reported to be effective in reducing the negative effects of stress [22-24]. Vitamin C functions to maintain the fat-soluble Vitamin E. In particular, Vitamin C also plays a role in activating Vitamin E when it loses its antioxidant capacity by turning into tocopherol. Vitamin C increases the antioxidant activity of Vitamin E by reducing chopheroxyl radicals into the active form of provitamin E [25]. A combination treatment of Vitamins C and E had been reported by several studies to reduce the oxidative stress in various conditions, that is, rats with diabetic pregnancy, heat stress in hen, and hypertensive rats [26,27].

Stress is a condition that can cause physiological disorders in the body through the response of ROS production. Stress can lead to weight loss, which occurs as a result of alterations in energy homeostasis regulation and reduced food intake [28-33]. Rats with CVS treated Vitamins C and E showed a significant increase in body weight. It was reported that Vitamin E supplementation could protect against changes in body weight and food intake due to stress [34].

In this present study, CVS resulted in increased SOD, MDA, GPx, and cortisol levels, while the CAT and testosterone levels were decreased. The fact that Vitamins C and E treatment in CVS rats resulted in the contrast suggests that the combination of Vitamins C and E administered improved antioxidant enzymes ability, endocrine function, and reduce lipid peroxidation in oxidative stress. This result was similar to those of several previous studies [35,36]. Increased antioxidant enzyme activities may be considered as a protective mechanism against stress-induced free radical production and lipid peroxidation [37].

SOD is an important parameter for measuring oxidative stress levels [38]. SOD is an enzyme that acts as a cellular antioxidant by catalyzing the free radical anion superoxide (O_2_^−^) to produce hydrogen peroxide (H_2_O_2_) and water (H_2_O), which is, in turn, degraded by CAT or GPx [39]. Electron transport disorders can be caused by stress through increased free radicals. Free radicals are unpaired molecules that are very reactive. Free radicals can change proteins, nucleic acids, and fatty acids in cell membranes and plasma lipoproteins [40]. Polyunsaturated fatty acid in cell membranes is easily reduced by free radicals through lipid peroxidation to produce MDA [37]. Lipid peroxidation can be improved by administering antioxidants. Lipid peroxidation is generally divided into three stages, that is, initiation, propagation, and termination. In the termination stage, antioxidants transfer hydrogen atoms, thereby reducing the reactive potential of the non-radical compounds [41]. The combination of Vitamins E and C reported reduces lipid peroxidation *in vitro* and *in vivo* [42]. The results of the present study are in accordance with the previous studies, which report that the combination of Vitamins C and E can reduce MDA levels in blood plasma [18,43].

In addition, CAT is one of the most important antioxidant enzymes that use hydrogen peroxide as its substrate and maintains cellular redox homeostasis [39]. Although the levels CAT in this study was decreased in CVS rats treated with Vitamins C and E, this is similar to the study in rats with restraint stress. Furthermore, the combination treatment of Vitamins C and E in rats resulted in an increase in CAT activities [44]. This increase might be due to the expression of CAT, which is induced by many kinds of stresses, including peroxide stress [45].

Stress leads to an imbalance in physiological responses associated with the activation of the hypothalamo-pituitary-adrenocortical (HPA) axis. Activation of the HPA axis results in an elevation in circulating glucocorticoids (cortisol and corticosterone). In particular, glucocorticoids are the main markers of stress [46,47]. Increased cortisol level during stress was reported in wilds, farms, domestic, and laboratory animals, such as primates, giraffe, chicken, fish, dog, and rat [48-53]. In the present study, the cortisol level was decreased when CVS rats were treated with Vitamins C and E. This finding was similar to a study involving stressed fish, in which increased cortisol levels did not result in depletion after administering Vitamins C and/or E as a supplement [52].

In contrast to the cortisol level, testosterone level was decreased during the stress condition. Stress affects the reproductive function through the activation of the HPA axis. HPA activation reduces the efficiency of the hypothalamus-pituitary-gonad axis [54,55]. Administering Vitamins C and E showed increased testosterone level in CVS rats, as well as in rats with testicular damage [56]. In addition, Vitamins C and E supplementation to diabetic rats resulted in the greatest improvement of the seminal parameters, such as sperm count, percentage sperm cell motility, percentage activity of motile cells, and percentage of cells with normal morphology [35].

## Conclusion

This present study showed that Vitamins C and E were effective in increasing the SOD, MDA, GPx, and cortisol levels in albino rats with CVS. In addition, there were increasing levels of testosterone. This study also provided evidence that Vitamins C and E modulate the percentage increase in body weight. The increase in body weight was followed by an increase in the levels of SOD, MDA, GPx, and cortisol. Furthermore, the increase in body weight was also followed by an increase in testosterone levels.

## Authors’ Contributions

MTEP supervised the study. NH and AP conducted the study. MTEP helped in the statistical analysis of the data. NH and FF helped in the preparation of tables, revised, and submitted the manuscript. All authors read and approved the final manuscript.
